# *Cyphostemmacalcarium*, a new species of Vitaceae from the Ankarana Special Reserve, Madagascar

**DOI:** 10.3897/phytokeys.180.69194

**Published:** 2021-08-03

**Authors:** Romer Narindra Rabarijaona, Valisoa Louisicaël Rafaralahy, Charles Rakotovao, Rindra Manasoa Ranaivoson, Bing Liu, Zhi-Duan Chen, Jun Wen, Li-Min Lu

**Affiliations:** 1 State Key Laboratory of Systematic and Evolutionary Botany, Institute of Botany, Chinese Academy of Sciences, Beijing 100093, China Institute of Botany, Chinese Academy of Sciences Beijing China; 2 University of Chinese Academy of Sciences, Beijing 100049, China University of Chinese Academy of Sciences Beijing China; 3 Department of Plant Biology and Ecology, Faculty of Sciences, University of Antananarivo, Madagascar University of Antananarivo Antananarivo Madagascar; 4 Missouri Botanical Garden, Madagascar Research and Conservation Program, BP 3391, Antananarivo 101, Madagascar Missouri Botanical Garden Antananarivo Madagascar; 5 Sino-Africa Joint Research Center, Chinese Academy of Sciences, Wuhan 430074, China Sino-Africa Joint Research Center, Chinese Academy of Sciences Wuhan China; 6 Department of Botany, National Museum of Natural History, MRC166, Smithsonian Institution, Washington, D.C. 20013-7012, USA National Museum of Natural History Washington United States of America

**Keywords:** Ankarana, *Cyphostemma*, *Cyphostemmacalcarium*, Madagascar, Vitaceae

## Abstract

*Cyphostemmacalcarium* Rabarij & L.M.Lu, **sp. nov.**, is herein described as a new species found on limestone outcrops in northern Madagascar. Its diagnostic morphological characteristics were compared to the species occurring in the Ankarana Special Reserve. We present detailed descriptions, illustrations, distribution map, and a preliminary conservation assessment of the species. An identification key to all known species of *Cyphostemma* from the Ankarana Special Reserve is also provided.

## Introduction

The genus *Cyphostemma* (Planch.) Alston contains ca. 200 species, representing the second largest genus following *Cissus* within the grape family, Vitaceae (Wen et al. 2018; Rabarijaona et al. 2020). Species of *Cyphostemma* are distributed mainly in Africa with a few species occurring in southern India, Thailand and southwest China (Dang et al. 2017; Wen et al. 2018). The genus is distinguished by several unique morphological characters: floral buds constricted at the middle, a floral disc of 4-large free glands, conspicuous stipules, and seeds with extra layers of endotestal sclereids covering the ventral infolds in cross-section (Wen 2007; Chen and Manchester 2007, 2011).

In Madagascar, *Cyphostemma* consists of ca. 25 species and displays substantial morphological diversity (Baker 1887; Descoings 1967; The Madagascar Catalogue Project 2019). Species are found in a diversity of habitats, including rainforests, savannas, dry thickets, dunes, and seasonal arid habitats such as the vegetation on limestones or an area within the reserve referred to as “Tsingy”. Several of the species of Vitaceae from Madagascar exhibit features that are very unusual in the family, such as succulent shrubs or trees, rather than lianas, and the lack of leaf-opposed tendrils (Hearn et al. 2018). Northern Madagascar possesses the highest species diversity for Vitaceae across the island, with ca. 68% at the family level and ca. 56% for *Cyphostemma* (The Madagascar Catalogue Project 2019).

**Figure 1.**
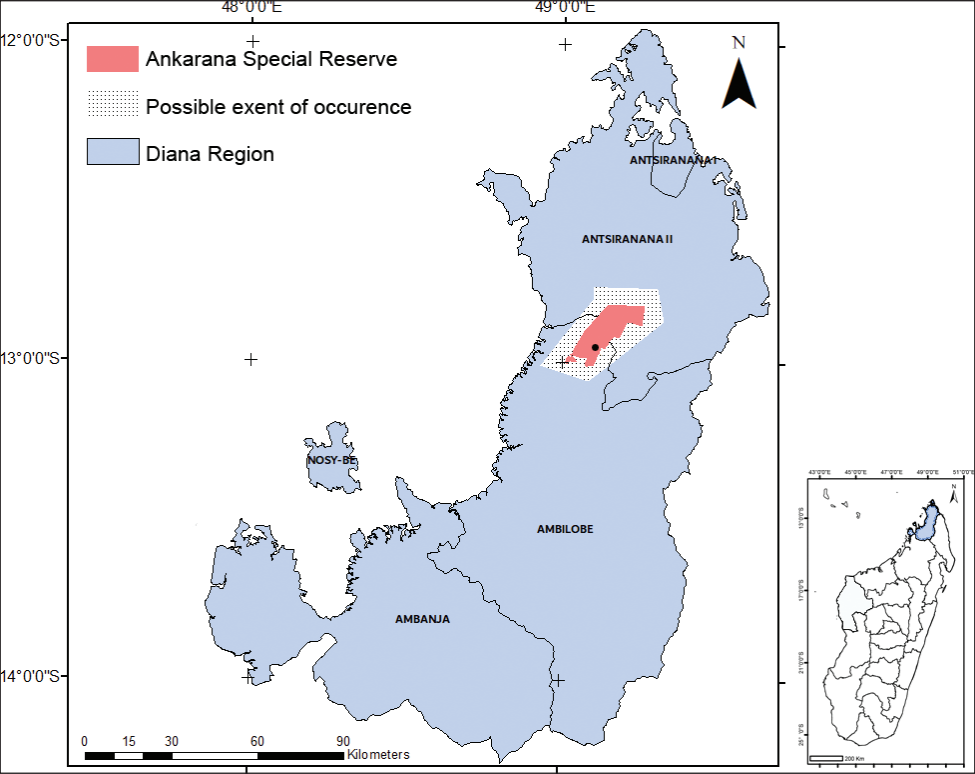
Distribution map of *Cyphostemmacalcarium* sp. nov. with the black dot showing the locality of the type specimens. Map on the right shows the position of Ankarana Special Reserve in Madagascar.

Since *Cyphostemma* species were reported to exhibit distinct morphotypes during their vegetative and flowering stages, we conducted an in-depth morphological investigation of all 25 described *Cyphostemma* species from Madagascar. Of the eight species of *Cyphostemma* in the Ankarana Special Reserve (Fig. 1), *Cyphostemmaankaranense* Desc., *C.caerulans* Desc., *C.rutilans* Desc., and the newly described species in this paper, all lack tendrils. However, the new species can be distinguished from the other three species based on several traits such as habit, stipule shape and size, leaf architecture, flower color, style length, and fruit shape (Table 1). We herein describe and illustrate this new species, assess its conservation status, and provide an identification key to all the species found in the Ankarana Special Reserve.

**Table 1.** Morphological comparison of four shrubby tendril-less species of *Cyphostemma* in the Ankarana Special Reserve, Madagascar.

Taxon	Habit	Stipule	Branch and leaf	Leaf architecture	Flower colour	Style length (mm)	Fruit
***C.ankaranense* Desc.**	suberect or prostrate	ovate to widely triangular, 12–25 × 6–10 mm	glabrous	bi-ternate to bi-pinnate	reddish	± 0.7	ellipsoid; 5–7 × 4–5.5 mm
***C.caerulans* Desc.**	prostrate	± falcate, 10–15 × 2.5–3.5 mm	glabrous	bi-pinnate	yellowish	± 1.5	globose or subglobose; 6.5–9 mm in diameter
***C.rutilans* Desc.**	erect	triangular; up to ca. 5 × 3 mm	glabrous	3-foliolate	reddish	± 1.5	ellipsoid; 6–8 × 5–6 mm
***C.calcarium* Rabarij & L.M.Lu**	erect	triangular to ± falcate; 4–5 × 1.5–2.5 mm	pubescent	3-foliolate, central leaflet often dropped	reddish	± 2.5	ellipsoid; 9–12 × 5–7 mm

## Materials and methods

The morphological description is based on measurements of dried specimens, supplemented by photos of mature living plants collected from the field. Herbarium specimens and digital images of the most closely-related species to the new described species were examined from the following herbaria: K, P, PE, and TAN. Protologues of type specimens were gathered from Descoings (1967) and JSTOR Global Plants (http://plants.jstor.org). Flowers, fruits, and seeds were dissected after briefly soaking in hot water. Images of floral parts and seeds were captured using a stereomicroscope (Leica DVM6 camera, Wetzlar, Germany). Terminologies describing seed morphology followed Chen and Manchester (2011).

## Taxonomic treatment

### Cyphostemma calcarium

Taxon classificationPlantaeVitalesVitaceae

Rabarij & L.M.Lu sp. nov.

3C656A32-3BDC-5D57-A359-175EE0BFD491

urn:lsid:ipni.org:names:77218853-1

Figs 2 , 3

#### Diagnosis.

*Cyphostemmacalcarium* is most closely comparable to *C.rutilans* Desc. in morphology. It differs from the latter in having distinct pubescent branches (vs. branches entirely glabrous in *C.rutilans*); leaves minutely puberulous and shiny on the adaxial surface, abaxial surface densely whitish pubescent to velvety particularly on the veins (vs. leaves entirely glabrous and shiny on both sides in *C.rutilans*); and leaflets broadly oblong or elliptic, base cuneate (vs. leaflets narrowly ovate, base subcordate in *C.rutilans*). Seeds of *C.calcarium* are ellipsoid in outline, 7–7.5 × 3–3.5 mm, surface rugose to ± muricate (vs. seeds globose, 5–6.5 mm in diameter, surface strongly rugose in *C.rutilans*).

**Figure 2.**
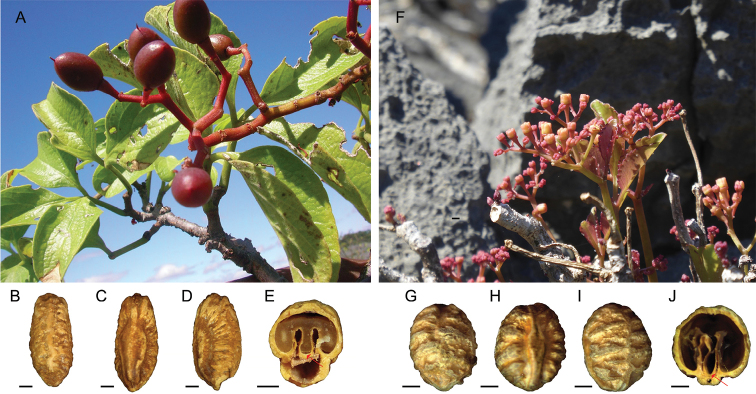
Comparison of two shrubby *Cyphostemma* species with 3-foliolate leaves in the Ankarana Special Reserve **A–E***Cyphostemmacalcarium* sp. nov. **A** branches showing puberulent leaves and infructescence **B–E** seed morphology from *Rakotovao C. et al.* 6376 (Dorsal, ventral, lateral, and cross-section presented from left to right) **F–J***Cyphostemmarutilans* Desc. **F** branches with glabrous leaves and inflorescence **G–J** seed morphology from Bardot-Vaucoulon M. 817 (Dorsal, ventral, lateral, and cross-section presented from left to right). Photos by Rakotovao Charles, Missouri Botanical Garden (**A**); Billiet Frieda, Meise Botanic Garden (**F**). The red arrow indicates an extra layer of endotestal sclereids covering the ventral infolds. Scale bars: 1 mm

#### Type.

**Madagascar.** Antsiranana: Diana, Ankarana Special Reserve, Tsingy Rary, 12°56'24.00"S, 49°07'04"E, 97 m, 16 May 2013, *Rakotovao C. et al.* 6376 (holotype: TAN!).

#### Description.

Succulent erect shrub, up to 2 m tall. Old stems swollen, succulent; bark smooth, lenticellate; branches brown to reddish, shortly pubescent. Tendrils absent. Stipules triangular to ± falcate, 4–5 × 1.5–2.5 mm, soon caducous. Leaves 3-foliolate, central leaflet often drooping, somewhat thick and fleshy when fresh, becoming coriaceous when dry, usually folded upwards along the midrib; leaflets 3–5 × 1.5–2.5 cm, broadly oblong or elliptic, base cuneate, rounded to obtuse at the apex, margin shallowly denticulate; minutely puberulous and shiny on the adaxial surface, abaxial surface densely whitish pubescent to velvety particularly on the veins; venation closely reticulate, prominent. Petioles 1.5–2.5 cm long. Petiolules equal, up to 1 cm long. Inflorescence a compound dichasium, terminal, ca. 7.5 cm long, very shortly pubescent; bracts inconspicuous; pedicels 2–4 mm. Floral buds ± 2.5 mm long, minutely puberulous or glabrescent; sepals ± 0.5 mm long; petals reddish; stamens 4, filaments cylindrical, ca. 2.2 mm long, anthers ca. 0.8 mm long; ovary glabrous, styles ± 2.5 mm long. Fruits ellipsoid, 9–12 × 5–7 mm, glabrous. Seeds broadly ellipsoid, 7–7.5 × 3–3.5 mm, rugose; base rostrate; beak conspicuous; apex revolute; rugae apex shallowly conspicuous on both surfaces; chalaza linear, sinuate, up to 6 mm long (ca. 6/7 of seed length); ventral ridge raised, elongate but widened in the middle, extending up to 6/7 of seed length; endosperm m-shaped in cross-section.

**Figure 3.**
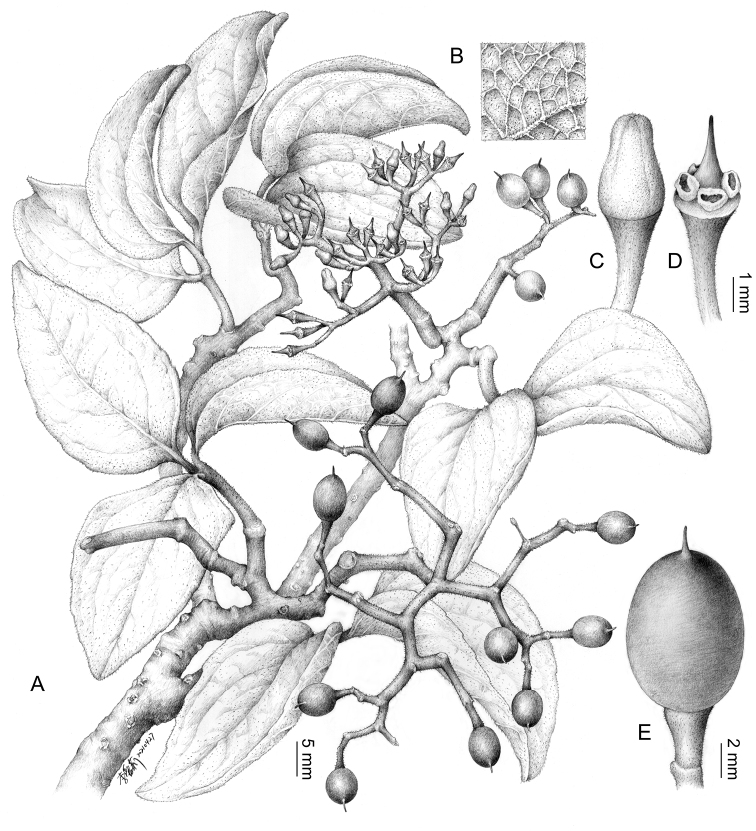
*Cyphostemmacalcarium* sp. nov. **A** branches showing the inflorescence and infructescence and the bark with distinct lenticels **B** trichomes on the abaxial leaflet surface **C** flower bud constricted at the middle **D** flower with petals and stamens removed to show the floral disc of 4-large free glands **E** fruit with a persistent stigma (Illustration by Ai-Li Li; based on *Rakotovao C. et al.* 6376, TAN).

#### Phenology.

Flowering and fruiting around May.

#### Etymology.

The epithet of the species refers to the habitats on limestone outcrops.

#### Distribution and habitat.

It grows on limestone outcrops in northern Madagascar at an altitude of 90–300 m. (Fig. 1)

#### Provisional conservation assessment.

The new species is endemic to Madagascar with distribution restricted to its type locality. It is assessed here as Critically Endangered (CR) according to the IUCN Categories and Criteria (IUCN 2019). Even though the species occurs within a protected area, succulent plants are still highly sought after by collectors for their horticultural values. Seeds of *Cyphostemmacalcarium* should therefore be collected, banked, and propagated to ensure its long-term conservation.

#### Taxonomic notes.

This species is described from materials collected by *Rakotovao C. et al*. in 2013. It was initially identified as *Cissuspileata* Desc., but it clearly belongs to *Cyphostemma* in having constricted flower buds and floral disks with four free glands. These characters, together with its M-shaped endosperm as viewed in cross sections of the seeds and the presence of extra layers of endotestal sclereids covering the ventral infolds in cross-section, clearly distinguish the new species from *Cissus* L. A summary of some diagnostic characters that differentiate this new species from other shrubby species of *Cyphostemma* in Ankarana Special Reserve is provided in Table 1.

### Key to the species of *Cyphostemma* in Ankarana Special Reserve, Madagascar

**Table:** 

1a	Shrubby succulent plants; tendrils absent	**2**
1b	Climbers to woody vines, sometimes tree-like; tendrils usually present	**5**
2a	Stems erect or suberect; leaves usually 3-foliolate; flowers reddish	**3**
2b	Stems rather prostrate; leaves pinnately arranged; flowers green to yellowish	**4**
3a	Young stems, branches, and petioles glabrous; leaves entirely glabrous and shiny on both sides; leaflets narrowly ovate, base subcordate	***C.rutilans***
3b	Young stems, branches, and petioles puberulent; leaves minutely puberulous and shiny on the adaxial surface, abaxial surface densely whitish pubescent to velvety particularly on the veins; leaflets elliptic, base cuneate	***C.calcarium***
4a	Leaflets narrowly oblong-elliptic, overall with a reddish tone; stipules ovate to widely triangular, 12–25 × 6–10 mm; flowers pale green; fruits ovoid or elongate–ellipsoid, apiculate	***C.ankaranense***
4b	Leaflets rhomboid, ovate or suborbicular, rather green; stipules ± falcate, lanceolate-acuminate, 10–15 × 2.5–3.5 mm; flowers yellowish; fruits globose or subglobose, not apiculate	***C.caerulans***
5a	Leaves digitately arranged, 3–5-foliolate	***C.glanduloso-pilosum***
5b	Leaves pinnately arranged	**6**
6a	Trunk sub-spherical, 0.50–0.70 m diameter; bark flaking, corky to reticulately fissured; inflorescences and flowers reddish	***C.pachypus***
6b	Trunk tree-like, up to 5 m tall or even taller; bark smooth, peeling, papery; inflorescences and flowers green to yellowish	**7**
7a	Leaflets 1-pinnate, densely pubescent; petioles 4–6 cm long; inflorescences 6–10 cm, usually shorter, densely pubescent; fruits subglobose, 12–13 mm in diameter	***C.macrocarpum***
7b	Leaflets 2-pinnate, glabrous; petioles 6–12 cm long; inflorescences 8–15 cm long, glabrous above, with scattered white-pubescence at the base of the nerves beneath, somewhat-like domatia; fruits ellipsoid, 10–12 × 6–7 mm	***C.greveanum***

## Supplementary Material

XML Treatment for Cyphostemma calcarium
